# Mediterranean-Type Dietary Pattern and Physical Activity: The Winning Combination to Counteract the Rising Burden of Non-Communicable Diseases (NCDs)

**DOI:** 10.3390/nu13020429

**Published:** 2021-01-28

**Authors:** Greta Caprara

**Affiliations:** Department of Experimental Oncology, IEO, European Institute of Oncology, IRCCS, 20139 Milano, Italy; greta.caprara@ieo.it; Tel.: +39-02-94375023

**Keywords:** Mediterranean diet, physical activity, exercise, non-communicable diseases, healthy lifestyle, life expectancy

## Abstract

Non-communicable diseases (NCDs) (mainly cardiovascular diseases, cancers, chronic respiratory diseases and type 2 diabetes) are the main causes of death worldwide. Their burden is expected to rise in the future, especially in less developed economies and among the poor spread across middle- and high-income countries. Indeed, the treatment and prevention of these pathologies constitute a crucial challenge for public health. The major non-communicable diseases share four modifiable behavioral risk factors: unhealthy diet, physical inactivity, tobacco usage and excess of alcohol consumption. Therefore, the adoption of healthy lifestyles, which include not excessive alcohol intake, no smoking, a healthy diet and regular physical activity, represents a crucial and economical strategy to counteract the global NCDs burden. This review summarizes the latest evidence demonstrating that Mediterranean-type dietary pattern and physical activity are, alone and in combination, key interventions to both prevent and control the rise of NCDs.

## 1. Introduction

Non-communicable diseases (NCDs), also known as chronic diseases (cardiovascular diseases, tumors, chronic respiratory diseases, type 2 diabetes, neurological pathologies, etc.) are the leading causes of premature death globally, responsible, every year, for the death of 41 million people. More than 70% of all deaths worldwide can be attributed to NCDs, eventually resulting in a huge economic impact [1]. Thus, the prevention and control of these pathologies is both a moral and an economic imperative.

As documented by several studies, unhealthy diets, physical inactivity, tobacco use and excessive alcohol consumption are the main risk factors causing the escalation of these pathologies. Nevertheless, since these detrimental behaviors are all modifiable, the increase in NCDs can be reduced simply changing our lifestyles [2,3,4,5,6,7].

This review will provide an overview of the positive effects exerted on health by a Mediterranean-type dietary pattern and a regular moderate physical activity (PA). The origins, main characteristics and outcomes on NCD prevention and treatment of the classical Mediterranean diet (MeD) will be discussed. Then, the effects on health and the feasibility of nutritional habits in countries beyond the Mediterranean region will be inspected. In addition, the main knowledge on PA, its different classifications and specific outcomes on physiology and metabolism will be reviewed. Finally, the combined effect of a Mediterranean type dietary pattern and PA on health will be described.

## 2. The Worldwide Status of Non-Communicable Diseases (NCDs)

Non-communicable diseases are responsible, every year, for 71% of all deaths globally: specifically, cardiovascular diseases (CVDs) account for most NCD deaths (31%), followed by tumors (16%), respiratory diseases (7%) and diabetes (3%) [1]. People of all age groups, regions and countries are affected by these illnesses: the 26% of all deaths attributed to NCDs occur between the ages of 30 and 69 years, with over 85% rising in low- and middle-income countries [1]. These conditions are often age-related, therefore, the fact that the world’s older population is progressively growing at an unprecedented rate renders this situation even more worrying. In fact, according to the report “Global strategy and action plan on ageing and health“, by 2050, one in five people will be 60 years or older [8].

Currently, NCDs are treated pharmacologically: hence, a progressive increase in patients, mainly elderly, affected by multiple chronic conditions, will result in less and less sustainable socioeconomic costs, making the prevention and control of these pathologies a crucial need [9,10,11].

Despite that the NCD rise is due to the combination of genetic, physiological and environmental factors, behavioral features, including unhealthy diets, physical inactivity, tobacco use and excessive alcohol consumption, are considered the primarily responsible for NCD escalation. In order to counteract the burden of NCDs, the World Health Organization (WHO) has established several global action plans and guidelines, which are mainly focused on educating people to pursue healthy habits [7].

Together with the NCD burden, the identification of sustainable and healthy food systems, able to adequately nourish, by 2050, an estimated global population of about 10 billion people, represents another issue the world needs to face, as soon as possible. The transition from an unhealthy to a healthy diet will allow reaching that aim, both feeding the future population and achieving the environmental sustainability goals [12].

## 3. The Mediterranean-Type Dietary Pattern

### 3.1. The Mediterranean Diet: Origin and Definition

A healthy diet is characterized by a correct caloric intake and is mainly focused on the consumption of plant-based foods; moreover, it favors the intake of unsaturated, rather than saturated, fats, low amounts of animal source foods and small amounts of refined grains, highly processed foods and free sugars [13,14,15,16]. All these features are consistent with many traditional eating habits, in primis the Mediterranean-type dietary pattern.

The MeD is a nutritional model arising from the food cultures of antique civilizations, which developed around the Mediterranean Basin (Greece, Italy, Morocco, Spain, etc.). Notably, even though the nutritional patterns prevailing among the populations of the Mediterranean Basin have many common characteristics, some differences in their dietary habits do exist, depending on several factors such as culture, climate and religion [17,18]. A study, which analyzed the foods habits of 16 Mediterranean countries [19], pointed out some diversities in the consumption of cereals, type of meat, fish, etc. For instance: (i) fish was more consumed in Morocco and in the South of Italy than in Turkey and Lebanon; (ii) pork was not consumed in the North African countries; (iii) pasta was mostly consumed in Italy, while couscous in the North African countries; (iv) pulses were most eaten in Spain, least in Malta; (v) olive oil consumption was abundant in Greece, rare in Egypt; (vi) some differences in the kind of vegetables and fruits eaten were also observed. However, although differentiated by foods, recipes and cooking methods, peculiar to each country, the MeD shares a common significant set of basic features and nutritional key compounds. Finally, the MeD (from the Greek “δíαιτα”, “díaita”, way of life) represents a set of knowledge, social habits and cultural traditions spread by the communities living in the areas surrounding the Mediterranean Sea.

The definition of a “Mediterranean diet” was coined around the 1950s, by the American physiologist Ancel Benjamin Keys, who first identified the link existing between the traditional diets of some Mediterranean populations and their low frequency of CVDs [20]. Subsequently, a number of studies confirmed the MeD benefits not only in preventing cardiovascular diseases, but also in counteracting other major NCDs [21,22,23].

### 3.2. The Mediterranean Diet: Nutritional Model

The MeD is mainly based on the regular consumption of extra virgin olive oil (as the main source of added fat) and plant-based food (whole grains, vegetables, legumes, fresh fruits, nuts, seeds, herbs and spices), a moderate amount of fish, seafood, dairy and poultry and a low consumption of red and processed meat, sugars, alcohol (mostly red wine) and pastry [18,24,25,26].

Regarding the distribution of macronutrients, the MeD contains approximately 55–60% of carbohydrates, with simple sugars representing less than 10%, 10–15% of proteins and 25–30% of fat (mostly monounsaturated—MUFAs—and polyunsaturated—PUFAs—whose main source is the extra virgin olive oil) [25]. Because of the major public health challenges of overweight and obesity, the serving sizes of MeD should be frugal and moderate, in order to adapt to the modern increasingly sedentary lifestyles [27]. Accordingly, mounting evidence has demonstrated that moderate calorie restriction, without malnutrition, exerts beneficial effects [28]. These characteristics are resumed in the “Mediterranean Diet Pyramid” (MeD Pyramid), an officially recognized worldwide nutritional guideline [27]. The portion sizes of the foods placed at the bottom of the pyramid should be larger and more frequently consumed, compared to those located at the upper levels. In fact, the former provide satiety, together with moderate amounts of energy, while the latter have higher fat and simple sugar contents [27].

The MeD Pyramid does not simply describe a balanced way of eating, displaying indication about the proportions and the frequencies of food consumption, but also includes conviviality, ecological and cultural elements, without forgetting the importance of being physically active and drinking an adequate amount of water [27].

### 3.3. The Mediterranean Diet: Effects on Health

Until the early 1900s, there was no proven evidence that diet could play a role in diseases prevention. Ancel Keys was the first to demonstrate that eating habits can influence the global health status and, in particular, the occurrence of cardiovascular disease. In 1957, he started the project known as “The Seven Countries Study”, showing the benefits of MeD in decreasing the risk of cardiovascular diseases and promoting the general healthspan [29,30,31]. This study comprised 16 cohorts of individuals, aged 40 to 59, from seven different countries (U.S., Finland, Netherlands, Italy [32], ex-Yugoslavia [33], Greece [34] and Japan), enrolling more than 12,000 persons [30,33]. The results of this work clearly showed that saturated fats consumption strongly correlated with severe coronary heart disease (CHD) and an increased risk of death. On the contrary, a high intake of MUFAs and flavonoids resulted in a lower incidence of ischemic heart disease mortality [35]. Altogether, these data clearly demonstrated, for the first time, that the traditional MeD was associated with a reduced risk of developing cardiovascular diseases [36].

In the last 50 years, other epidemiological and physio-pathological evidence confirmed the beneficial effects of MeD on health and longevity [37,38,39,40,41,42,43]. Several studies and meta-analysis of prospective cohort studies (including more than 1.5 million subjects) showed that a strict adherence to the MeD correlated with an improved health status, leading to a significant reduction in overall mortality (9%), mortality due to cardiovascular diseases (9%), tumor occurrence and progression (6%), and an incidence of Parkinson’s and Alzheimer’s disease (13%) [44,45,46,47,48,49].

#### 3.3.1. How to Measure the Adherence to the Mediterranean Diet in Clinical and Epidemiological Studies

As previously described, many studies have consistently demonstrated the protective role of MeD against the development of several NCDs. Nevertheless, this effect can vary between works, mainly because of the differences existing among the foods characteristics of each country [19]. Therefore, several “adherence scores” have been created, in order to better ascertain the degree of adherence to MeD. These measurement tools are based on the frequency of pattern-consistent and -inconsistent food consumption and on the compliance with the recommended intake. They are composite structures based on the combined measure of dietary components, foods and nutrients, in order to obtain variables that evaluate the association between the quality of diet and its effects on health [50].

Several MeD indexes have been identified, which differ for the number of components (7–28); the scoring (0, 1, 2, 3, 4, 5, 8 or 10, in case of compliance); the range of adherence (0–100) and type of components (food groups/foods or their combination with nutrients). Other differences were also represented by their composition and evaluation (for instance the criteria used to define “moderate alcohol consumption”) and the scoring system (medians, terciles, established servings) [51,52].

The “Mediterranean Diet Score” (MDS-1) was the first index created to study the adherence to the traditional Greek MeD [53]. Briefly, MDS-1 was constituted of eight components ((i) high MUFAs/SFAs ratio, a (ii) high cereals intake—including bread and potatoes—; (iii) high vegetables intake; (iv) high fruit and nuts intake; (v) high legume intake; (vi) moderate milk and dairy products intake; (vii) moderate alcohol intake; (viii) low meat and meat products intake) and it was based on assigning a score from 0 to 1 to the daily intake of each component. The medians of the sample, specific for sex, were used as cut-off points. If the intake of a component was over the sample median, for the protective components (fruits, vegetables, etc.), or if it was below the median, for the non-protective components (meat, dairy products, etc.), 1 point was assigned. In the opposite situations, 0 points were assigned. Thus, the MDS-1 might range from 0 (minimal adherence) to 8 (maximum adherence) (Table 1). Generally, a score of 4, or more, was associated with a satisfactory MeD adherence and better health implications [53,54].

The MDS-1 index is the most widely used score. However, more than 22 variants have been created to be used in different geographical populations [51,52,55]. Table 1 resumes the main characteristics of some MeD adherence scores. Many authors have described and reviewed, in detail, the studies that have established the MeD indexes and their relationship to NCDs development and mortality [50,51,56].

Importantly, not all the described indexes share the same features found in the original MDS-1: the major differences are related to the components and the scoring system. Some of these indexes, in fact, have been created to adapt the MeD to the population and countries not belonging to the Mediterranean Basin. The correct evaluation of indexes of adherence to MeD is crucial to establish their predictive capacity of disease risk: despite that, in several studies, the MeD–diseases relationship has been analyzed using many different indexes [52,56]. As a result, even though most of the MeD indexes were associated with the prevention of NCDs, sometimes others do not offer a strong predictive capacity concerning disease risk and mortality. Thus, the evaluation of the adherence to MeD and its relationship with disease prevention should be always carefully interpreted, paying attention to the indexes used in any study. To overcome the problems due to this heterogeneity, it is necessary to establish a common criterion to identify the components to be included in the definition of a Mediterranean-like dietary pattern, so that it would be possible to more accurately and evenly measure the adherence to this nutritional model and its relation to disease risk and mortality, in any population.

#### 3.3.2. Cardiovascular Diseases (CVDs)

Starting from Ancel Keys’ pioneering work, a number of studies have highlighted the protective and preventive effects of MeD on CVDs [67,68,69,70,71,72,73]. It has been demonstrated that extra virgin olive oil (a rich source of MUFAs and phenolic compounds with strong antioxidant and anti-inflammatory properties) together with walnuts, fish (rich in omega-3 fatty acids), fibers and phytosterols (with antioxidant activities) may have anti-atherogenic properties, contributing to the MeD cardioprotective actions [23,72,74,75,76,77]. Reliable evidence consistently supported that the adherence to the MeD is associated with better cardiovascular health outcomes, including reduced blood pressure levels and markers of vascular inflammation, together with a decreased rate of ischemic stroke and CHD [73].

More recently, an umbrella review of meta-analyses of randomized controlled trials, which evaluated the effects of different diets on cardiometabolic risk factors and anthropometric parameters, demonstrated that the MeD had the strongest and most consistent beneficial effects on both these aspects [78]. The main characteristics of the cited studies are described in Table 2.

Moreover, the adoption of a Mediterranean-like nutritional pattern has been recently shown to be important also in cardiac rehabilitation (CR), which is included, together with PA, in the secondary prevention strategies employed to reduce recurrent cardiovascular events and to improve the quality of life in patients with CVD [79,80]. Large cohorts of patients with a history of CVD, from the Health Professionals Follow-Up and the Nurses’ Health studies, were followed for a median of 7.7 years, for men, and 5.8 years, for women. The dietary information collected at the end of this study demonstrated that the adherence to a Mediterranean-like nutritional pattern was associated with a reduced risk of mortality [81]. Recently, another study, which included more than 15,000 patients with stable CHD, showed that following a MeD resulted in a reduced risk of cardiovascular death, non-fatal myocardial infarction and non-fatal stroke [82].

Importantly, the impact of dietetic interventions on the primary or secondary prevention of CVD is not the same. Nutritional intervention, in the context of primary prevention, focuses on the modification of cardiological primary risk factors (hypertension, overweight/obesity, dyslipidemia, etc.) in the general population, while in the secondary prevention the primary outcome is to reduce the risk of more serious and possibly fatal novel complications, in subjects which have already experienced a CV event. Moreover, secondary prevention programs are more structured and include other components, such as PA and drug therapy [83,84,85]. It is, therefore, not surprising that primary prevention studies, on the effect of MeD on CVD, outnumbered those on secondary prevention, the latter being more cost-effective and complicated to perform, interpret and analyze.

Indeed, further studies are required to better evaluate and quantify the efficacy of the MeD for secondary prevention of CVD.

An excess of salt consumption (higher than 5 g a day) has been related to hypertension and an increased risk of cardiovascular diseases, in particular heart disease and stroke [86]. Despite that, most people consume an average of the 9–12 g of salt/day. WHO recommends the consumption of less than 5 g/day of salt, estimating that 2.5 million deaths/year could be prevented as a result of this reduction [87]. The traditional Mediterranean eating pattern, poor in processed foods (particularly rich in salt), abundant in plant foods (containing potassium, which contributes to reduce blood pressure) and characterized by the usage of herbs and spices, rather than salt [88], might have beneficial cardiovascular effects. Probably because of the heterogeneity of MeD patterns within the different regions and countries, evidence for clinically important effects of Mediterranean diet and reduced salt is limited [89]. However, it has been extensively demonstrated that a DASH (Dietary Approaches to Stop Hypertension) diet, which is based on the MeD with a salt consumption lower than 4 g/day, is able to prevent and control hypertension, thus lowering the risk of CVD development [90].

Moderate alcohol consumption (≤1 drink/day for women and a maximum 2 drinks/day for men) is associated with a lower risk for CVD mortality. In particular, red wine has been linked with better long-term CVD outcomes [91,92]. This effect can be due to the presence of several phenolic compounds, such as resveratrol, having high antioxidant potential [92]. Interestingly, it has been demonstrated that the beneficial effects of red wine can be enhanced in the context of MeD, possibly as a consequence of the synergy with extra virgin olive oil and/or other foods rich in antioxidant compounds [93]. Importantly, very often, the average moderate wine drinkers are more likely to be of a higher educational and socioeconomic status following a healthy lifestyle [94]. Of note, most of the studies on the relationship between alcohol and health are observational and drinking more than 1 drink/day for women and 2 drinks/day for men increases the risks of CVDs and cancer (see below) [91,95]. Accordingly, health care professionals should not advise nondrinkers to consume alcohol in order to reduce CVDs risk.

#### 3.3.3. Tumors

The MeD also revealed strong preventive effects on several types of tumor. In particular, the “European Prospective Investigation into Cancer and Nutrition (EPIC)” epidemiological study highlighted that the Mediterranean nutritional model is the most effective in reducing the overall cancer risk [96]. The preventive influence of the Mediterranean lifestyle on the onset of tumors [58,97,98,99] is particularly robust on stomach [100,101,102,103], esophageal [104], colorectal [105,106,107,108,109], prostate [110,111,112], mammary and endometrial cancer [113,114,115,116,117,118].

The tumor-preventive effect of the MeD is probably due to the high contents of antioxidants and anti-inflammatory nutrients contained in many foods, such as legumes, fresh fruits, nuts, vegetables, fish and extra virgin olive oil, which is remarkably rich in phenolic compounds (oleuropein, hydroxytyrosol, tyrosol, etc.). These elements can exert a protective role against cancer development and progression, preventing DNA damage and reducing cell degeneration, proliferation and metastasis [99,119,120].

Importantly, the World Cancer Research Fund (WCRF) indicates that all kinds of alcoholic drinks can drive cancer formation, to an extent depending on the amount and frequency of alcohol consumed. The mechanisms explaining why alcohol consumption triggers tumor development is not completely understood yet: however, mounting evidence has shown that acetaldehyde, the principal metabolite of alcohol, may have a genotoxic effect, thus contributing to the carcinogenic cascade. In conclusion, as WCRF states that: “For cancer prevention, it’s best not to drink alcohol”, the recommendation for people is, at least, do not exceed the amount defined by the national guidelines (≤1 drink/day for women and a maximum 2 drinks/day for men) [95].

#### 3.3.4. Chronic Respiratory Diseases

Chronic obstructive pulmonary disease (COPD), the fourth leading cause of death worldwide, is a chronic pulmonary disease characterized by long-term breathing problems and progressive, not fully reversible, airflow limitation [121]. This pathology is strongly influenced by genetics, environmental and behavioral factors, including physical inactivity and diet. In the last twenty years, several studies have shown that the MeD, particularly rich in fruit, nuts, vegetables, fish and whole grains, is associated with a reduced risk of COPD [122,123,124,125]. This observation suggests that the amount of antioxidant compounds provided by the aforementioned components can reduce the occurrence of an oxidative microenvironment in the lung. Moreover, the anti-inflammatory properties of omega-3 fatty acids, as well as the pro-inflammatory properties of omega-6 fatty acids, might be crucial, considering the pivotal role played by inflammation in COPD progression [125]. Finally, the lower amount of processed meat might contribute to the reduction in an oxidative stress condition in the lung: of note, a recent meta-analysis demonstrated that each 50 g of the increased intake of processed meat per week was associated with an 8% increased risk of developing COPD [126]. Accordingly, it has been speculated that the high content of food additives in processed meat, including nitrites, can induce oxidative stress and inflammatory processes in lung cells [127].

#### 3.3.5. Type 2 Diabetes (T2DM)

MeD also demonstrates a beneficial effect in glycemic control, insulin sensitivity and the primary prevention of T2DM [128,129,130]. In particular, two epidemiological studies showed that MeD, which involved the consumption of carbohydrates with low glycemic load, is able to reduce the risk of diabetes by 20%, possibly by attenuating inflammation and oxidative stress [131,132]. The effects exerted by the MeD on T2DM could be attributed to the high antioxidant and anti-inflammatory properties of the whole dietary pattern, which are associated with decreased biomarkers of subclinical inflammation and increased levels of adiponectin, an anti-inflammatory cytokine inversely associated with T2DM risk [133,134]. In addition, the richness in dietary fiber is believed to induce satiety, reducing caloric intake, preventing weight gain [135] and thus exerting an indirect protective effect against the disease. Moreover, water-soluble gel-forming fibers, such as β-glucan, reducing the contact of macronutrients with digestive enzymes in the small intestine, cause a delay in glucose absorption, hence decreasing both fasting blood glucose and insulin concentrations [136]. Finally, the employment of unsaturated fatty acids (PUFAs and/or MUFAs) instead of saturated (SFAs) and trans fatty ones (TFAs) exerts beneficial effects on insulin sensitivity [137].

#### 3.3.6. Overweigh and Obesity

Obesity is a chronic disease associated with several pathological conditions, such as hypertension, dyslipidemia, cardiovascular diseases, alterations of glucose metabolism, T2DM, metabolic syndrome (MetS) [138] and cancers [5,139]. Several epidemiological studies clearly demonstrated that a firm adherence to the traditional MeD correlates with a reduction in body mass index (BMI), overweight, obesity and the onset of MetS [140,141,142,143]. Accordingly, the latest World Cancer Research Fund (WCRF) report indicates that MeD is a nutritional pattern able to maintain a healthy BMI, protecting against the development of 12 different kind of tumors [5]. Finally, a hypocaloric version of this nutritional approach, combined with regular PA, represents a safe strategy for an effective and stable weight loss [37,38,42,135,144].

#### 3.3.7. Osteoporosis

The adherence to MeD has been shown to be effective in reducing bone loss and osteoporosis [145,146,147,148]. The “PREDIMED” (Prevención con Dieta Mediterránea—Prevention with Medi-terranean Diet) trial, involving cardiopathic individuals between 55 and 80 years old, demonstrated that MeD was associated with a lower risk of osteoporotic fractures [149]. Some phenolic compounds found in the extra virgin olive oil (apigenin, luteolin, ferulic, p-coumaric and caffeic acid), have been suggested to be among the components responsible for the beneficial effects of the MeD on bone health. In particular, these compounds favor osteoblastic differentiation and maturation, by increasing the synthesis and activity of alkaline phosphatase and inducing calcium deposition in the extracellular matrix [150].

#### 3.3.8. Microbiota

Mounting evidence indicates that the microbiota exerts several beneficial effects on health, by enhancing the host’s response toward diseases, increasing the nutrient-exploitation capacity, neutralizing drugs and carcinogens, modulating intestinal motility and establishing an important feedback with the central nervous system (CNS) [151]. Indeed, a healthy microbiota is crucial to guarantee protection against several diseases, making the impact of diet on microbiota extremely important.

Interestingly, a strict adherence to MeD results in the presence, in the feces, of an elevated concentration of the microbial metabolites short chain fatty acids (SCFAs), derived from dietary fiber fermentation processes: SCFAs exert a recognized anti-inflammatory and antitumor effect, in particular against the development of colorectal cancer (CRC) [152]. Specifically, SCFAs (propionate, acetate, and butyrate) promote the growth of beneficial microbiota species (namely Bifidobacteria and Lactobacilli), exert anti-carcinogenic and anti-inflammatory activities, through the inhibition of GPR41-mediated NFκB transcription, and are able to regulate immune response in the intestine [152,153]. Some studies have demonstrated that butyrate is able to modulate gut homeostasis, controlling intestinal macrophage function: the treatment of macrophages with butyrate leads to the downregulation of Lipopolysaccharide (LPS)-induced pro-inflammatory mediators, including IL-6, IL-12, and Nitric oxide (NO) [154]. Similarly, phytochemicals from cruciferous vegetable exert an anti-inflammatory effect by modulating the aryl hydrocarbon receptor (AHR) activity, hence reducing colonic inflammation [155]. In addition, quercetin, a phytochemical mainly found in fruits and vegetables, was able to suppress, in vitro, LPS-induced and spontaneous inflammation in organoids from, respectively, wild type (WT) and ulcerative colitis mouse model [156]. Furthermore, a recent study showed that quercetin is effective in mice, even in recovering gut microbiota after antibiotic treatment, thus suggesting its possible prebiotic effect on gut microbiota [157]. Finally, omega-3 PUFAs are able to modulate gut homeostasis, preventing inflammatory responses within the gastrointestinal tract. Specifically, omega-3 PUFAs can inhibit the transcription of pro-inflammatory mediators, activate anti-inflammatory responses [158,159], promote the resolution of inflammation, stimulate macrophage phagocytosis and reduce the secretion of pro-inflammatory cytokine via specialized pro-resolving mediators (SPMs) [160,161,162]. Of note, SPMs, which are derived from the metabolism of omega-3 PUFAs, are being tested in several clinical trials, showing beneficial effects in inflammatory bowel disease patients [161,162,163,164,165].

Overall, the nutritional Mediterranean pattern leads to lower serum levels of inflammatory markers, including pro-inflammatory cytokines, adhesion molecules and chemokines, guaranteeing an anti-inflammatory response in the gut and beneficially impacting both microbiota and host health [166,167].

In contrast, individuals with low adherence to the Mediterranean nutrition model show a significant concentration, in the urine, of N-oxide of trimethylamine (TMAO), a microbial product associated with the development of atherosclerosis, cardiovascular disease and CRC. Moreover, imbalanced diets, rich in refined grains, sugars, saturated fatty acids, red and processed meat, as opposite to the Mediterranean nutritional pattern, favor the increase in chronic inflammation within the intestine, that might finally result in several NCDs, including CRC development and progression [166,167]. In addition, microbiota dysbiosis has been frequently observed, influencing the host’s immune system through several mechanisms, such as the modification of the signaling via the NLRP6 inflammasome and Toll-like receptors (TLRs), the reduction in adenosine monophosphate (AMP) and mucus release into the lumen, the degradation of secretory immunoglobulins A (IgAs) and the selective loss of IL-10-producing Treg lymphocytes [168,169]. This results in the perturbation of barrier integrity and the alteration of intestinal immune cell homeostasis [170,171]. For instance, it has been shown that a high intake of SFAs modifies the gut microbiota composition, increasing the proportion of Gram negative bacteria and altering the intestinal permeability, which finally leads to a state of metabolic endotoxemia [172]. Moreover, SFAs are also able to activate a pro-inflammatory response, in intestinal macrophages, through the stimulation of a TLR4-induced inflammatory pathway, which in turn triggers NF-κB and induces the expression of several pro-inflammatory mediators [173].

More recently, it has been demonstrated that the MeD, through the modulation of the gut microbiota, has the potential to promote healthier aging and reduce blood cholesterol levels in obese patients, independently of the energy intake [174,175].

#### 3.3.9. Overall Longevity

In agreement with the previously discussed effects on health, it is not surprising that MeD, together with a healthy lifestyle, can prolong life expectancy [176,177]. The regular intake of foods rich in antioxidant and anti-inflammatory molecules, in fact, can hinder not only the onset of several diseases, but also delay the aging process (reducing oxidative stress, inflammation and shortening of telomeres) and promote a healthy longevity [178,179]. Consistently, the Sicanian Mountains (Sicily), Cilento (Campania) and Sardinia, in Italy, together with Icaria, in Greece, are among the world areas where people live longer [180,181].

In order to understand how MeD could contribute to the achievement of healthy aging, several studies have been performed [182,183]: in particular, “The Healthy Aging: a Longitudinal study in Europe (HALE)”, which aimed to evaluate the association of a specific diet and lifestyle with the mortality rate in the elderly population, which showed that, even at an age between 70 and 90, following a MeD in the context of a healthy lifestyle reduces all causes of death by more than 50% [184]. More recently, a one-year European randomized dietary intervention, the “NU-AGE (New Dietary Strategies Addressing the Specific Needs of the Elderly Population for Healthy Aging in Europe) Randomized Trial”, including 1296 European men and women aged 65–80 years, showed that the MeD was able to ameliorate bone density and improve cognitive health [148,185,186,187].

#### 3.3.10. Mediterranean Diet and Epigenetic Changes

Epigenetics is defined as heritable changes in gene expression that are not attributable to alterations in the DNA sequence. Epigenetic marks modify the chromatin environment, affecting DNA accessibility and regulating a wide range of processes, including gene transcription. The major epigenetic mechanisms are represented by DNA methylation, histone modifications (methylation and acetylation) and non-coding RNA [188,189]. Unlike the DNA sequence, epigenetic marks can undergo changes in response to several stimuli, including environmental exposures such as nutrients, pesticides, toxins and pollutants.

Notably, several components of the MeD contain bioactive compounds of particular interest in the field of epigenetics, especially for their anti-tumor activity, [190]. The polyphenolic compound resveratrol, found in grapes, some berries and peanut oil seeds, is able to modulate the DNA methylation status of several genes involved in cancer, hypomethylating and hypermethylating key tumor-suppressors and tumor-promoters, respectively [191,192]. The antioxidant carotenoid lycopene, naturally present at high concentrations in tomatoes and, to a lesser extent, in many fruits, has been shown to reduce prostate cancer risk through the regulation of the expression of serine/threonine kinase 2 (AKT2) and MicroRNA-let-7f-1 [193]. Anthocyanins, a group of pigments mainly found in berries, black grapes, aubergines, cruciferous vegetables and pomegranate, are able to influence cell cycle at an epigenetic level, promoting the mechanisms of DNA-repair [194]. In addition, fisetin, a flavonol found in strawberries, apples, persimmons, onions and cucumbers, have been shown to inhibit cancer growth, by targeting several signaling pathways, including the mTOR and PI3K/AKT cascade [195,196]. Many epigenetic mechanisms are also associated with the flavonol quercetin (found in red onion, cruciferous vegetables, tomatoes, berries, red grapes and citrus fruits), such as the suppression of the non-receptor tyrosine kinase janus kinase 2 (JAK2), which induces autophagy and apoptosis in cancer cells [197]. Sulforaphane, a sulfur-rich compound found in cruciferous vegetables, exhibits a well known epigenetic action via the inhibition of histone deacetylase enzymes [198].

Interestingly, adherence to the complete MeD has been associated with the epigenetic modifications of genes related to inflammation, immunocompetence and signal transduction. Understanding the meaning of these changes may help explain the protective effect of MeD against the development of CVDs, diabetes and metabolic diseases. A subset of 36 individuals at high cardiovascular risk was selected within the PREDIMED-Navarra study [72,199]: after 5 years of intervention, changes in the methylation status of peripheral blood cells were analyzed. Adherence to MeD was found to be associated with methylation and the potential regulatory impact of eight genes related to inflammation and immune response [200]. Moreover, specific components of MeD, such as nuts and extra virgin olive oil (both rich in MUFAs and PUFAs), were able to induce methylation changes in several peripheral white blood cell genes [201].

Interestingly, even age-associated epigenetic variations have been shown to be affected by diet. A very recent pilot study, performed on 120 elderly healthy subjects from the NU-AGE study, demonstrated that following a Mediterranean-like diet for 1 year, promoted an epigenetic rejuvenation of participants [202].

Despite several promising data [203], further studies are needed to better clarify the epigenetic mechanisms through which the bioactive compounds characteristic of the MeD exert their activity. This would be a key achievement, in order to understand how these peculiar nutritional elements can be more specifically employed in the prevention and treatment of NCDs.

### 3.4. The Mediterranean-Type Dietary Pattern in Countries beyond the Mediterranean Area

Considering the aforementioned epidemiological evidence, which demonstrated the huge health benefits exerted by the MeD, it is not surprising that, even in other areas of the world, this type of eating pattern has started to be studied and when feasible, recommended, in order to promote healthier food choices to counteract the occurrence of chronic diseases [204,205,206,207,208].

Indeed, the adoption of a traditional Mediterranean-like nutritional pattern can be very useful for Western populations (U.S., Canada, Mexico, United Kingdom, Australia and New Zealand, amongst others), whose poor diet quality has become the primary cause of obesity, chronic diseases and mortality [209,210].

Starting from 2005, in order to react to the burden of chronic diseases, the U.S. Department of Health and Human Services and the U.S. Department of Agriculture (USDA) have established the so called “Dietary Guidelines for Americans”, which are revised every five years. In particular, in the 2015–2020 Dietary Guidelines, the MeD was adapted to the American population and an alternative healthy Mediterranean-style eating pattern included [211]. In addition, the Harvard School of Public Health [212], the nonprofit organization Oldways [213] and other institutions offer practical resources to apply the traditional MeD to Americans. Particularly noteworthy is the Healthy Eating Plate, created by nutrition experts at the Harvard School of Public Health (HSPH). Based on dietary strategies that strongly remind the Mediterranean habits, the Healthy Eating Plate is a visual guide that complements the healthy eating pyramid [214] and represents a healthy way to assemble a balanced meal, helping people to make the best eating choices [212].

Interestingly, the effectiveness of a Mediterranean-like diet on U.S. population health has been largely demonstrated: in fact, several studies have shown that a Mediterranean-like diet favorably influences the lipid profile in postmenopausal women [215], decreases the risk of vascular events and death from all causes, including CVD and cancer [216,217,218], reduces the rate of cognitive decline with older age [219], mitigates MetS [220] and generally ameliorates the quality of life, decreasing pain, disability, and depressive symptoms [221]. However, more studies are needed, in particular long-term trials, in order to test the efficacy of a full Mediterranean food pattern on American health. To this extent, “The National Heart, Lung, and Blood Institute” is planning to fund a trial called “Testing the Effects of a Mediterranean Dietary Pattern on Cardiovascular and Other Diseases in the United States” [205].

In addition, prospective and cross-sectional studies performed by Australian researchers also confirmed several benefits of a Mediterranean-style diet, including a reduction in cardiovascular risk, [222], a decreased risk of diabetes-induced mortality and enhanced glycemic control in diabetes patients [223,224], together with improved mental health and reduced depressive symptoms [225].

Moreover, greater adherence to the MeD was also associated with lower CVD incidence and mortality in a UK cohort and in large Eastern European urban populations (Czech Republic, Poland and the Russian Federation) [226,227].

These data, in addition to other similar findings, clearly paved the way for additional future population-based and clinical studies to determine the effectiveness of a MeD pattern in contemporary, non-Mediterranean populations. However, it is essential to consider that it would not be easy and/or always possible to adapt a Mediterranean-type dietary pattern to the inhabitants living outside this geographical area.

#### Feasibility Issues

Although there is extensive epidemiological evidence supporting the health benefits of a MeD, some studies conducted outside the Mediterranean Basin faced several barriers, in term of culture, economy, sociality, food availability (in particular extra virgin olive oil), costs, etc. [228,229,230]. Scientists are working to tackle those issues, in order to find new approaches to promote healthy dietary behaviors, consistent with the MeD. Moreover, it is crucial to identify a uniform evaluation score to define the adherence to MeD in epidemiological studies and detect the key compounds and molecular mediators responsible for its beneficial effect. In the meanwhile, it is worth trying to adapt the Western diets to the major healthy principles of the traditional MeD, as outlined in Table 3.

Further studies are needed to understand whether a Mediterranean-type dietary pattern is feasible for some low- and middle-income countries, such as many South Asian countries (for instance Nepal and India) and Africans regions, as well as Nations with diets and lifestyles very different from the Western ones (as China). While waiting for those answers, the consumption of a diet that not only provides the body with essential nutrients (fluids, macronutrients, micronutrients and adequate amount of calories), but which is also scientifically certified as healthy and sustainable for the planet (meaning containing whole grains, fresh fruits, vegetables, good quality proteins—pulses, fish, poultry, dairy products, eggs—and a moderate amount of red meat, little to no processed and packaged foods, sweets and sweetened beverages) should be globally encouraged.

For instance, the New Nordic Diet (NND) represents an example of a healthy alternative to the Mediterranean dietary pattern and it is considered a paradigm of sustainable and healthy diet integrating cuisine and dietary habits from the five Nordic countries [231]. Nevertheless, the NND shares many nutritional elements with the traditional MeD: in particular, (i) it is rich in plant-based foods (fresh fruits, vegetables, legumes, whole grains, aromatic herbs, mushrooms, seaweeds); (ii) it mainly employs MUFAs and PUFAs (from fish, nuts, seeds and canola oil—instead of extra virgin olive oil—); and (iii) red meat, processed foods and sweets are rarely consumed [14].

In addition to NND, the traditional Japanese and Okinawa diets have been associated with a reduced incidence of many chronic diseases (CVDs, some tumors, T2DM, etc.) and mortality. Interestingly, despite being characteristic of an Asian country, Japanese and Okinawa diets share many important features with the traditional MeD, such as: (i) the high intake of unrefined carbohydrates; (ii) low glycemic load; (iii) high consumption of phytonutrients and antioxidants compounds; (iv) healthy fat profile (high in MUFAs and PUFAs, low in SFAs and rich in omega-3 PUFAs); (v) low caloric intake and a moderate protein intake, mainly of plant and fish origin [232].

This evidence shows that, even though an accurate reproduction and application of the MeD might not be feasible in every country, the adoption of a dietary pattern, sharing major nutrients and compounds characteristic of MeD, should be encouraged and promoted, while respecting local cultural and socio-economic traditions.

## 4. Physical Activity

The US Centers for Disease Control and Prevention (CDC) defines PA as “any bodily movement produced by the contraction of skeletal muscle that increases energy expenditure above a basal level. Physical activity generally refers to the subset of physical activity that enhances health.” Exercise, instead, is defined as “A subcategory of physical activity that is planned, structured, repetitive, and purposive in the sense that the improvement or maintenance of one or more components of physical fitness is the objective” [233]. Regardless of those definitions, the WHO claims that any kind of PA can provide health benefits, not only when undertaken regularly and of sufficient duration and intensity (walking, cycling, dancing, doing sports, etc.) but also when it is carried out as part of work (lifting, carrying, etc.) or domestic tasks (cleaning, carrying, washing, etc.) [234].

### 4.1. The Adverse Outcomes of a Sedentary Behavior

The positive effect exerted by a moderate PA on health was already recognized, in the 5th century BC, by the physician Hippocrates who stated: “All parts of the body, if used in moderation and exercised in labors to which each is accustomed, become thereby healthy and well developed and age slowly; but if they are unused and left idle, they become liable to disease, defective in growth and age quickly.” Nowadays, scientific evidence has consistently demonstrated this assertion, highlighting how physical inactivity represents one of the leading causes of overweight, obesity and NCDs, resulting in the fourth leading risk factor for global mortality [235,236].

Although recent big technological changes have improved and simplified our lives, on the other hand, the massive urbanization, the development of automation technologies and changing patterns of transportation have led to a drastic decrease in PA, driving population towards increasingly sedentary behaviors. Indeed, several studies have demonstrated a severe reduction in the overall PA levels in the last 40 years. Globally, it has been estimated that one out of four adults and three out of four adolescents (aged 11–17 years) are insufficiently physically active, meaning that they engaged in less than 150 min of moderate intensity PA per week and less than 60 min of moderate to vigorous intensity PA daily, respectively [234,237,238]. Indeed, it is not surprising that worldwide obesity has nearly tripled since 1975: in 2016, more than 1.9 billion adults, 18 years and older, were overweight and, among these, over 650 million were obese [239]. As for NDCs, overweight and obesity, once considered a high-income country problem, are now on the rise in low- and middle-income regions, particularly in urban settings, becoming currently recognized as extremely severe public health issues [239]. As already discussed, in fact, high BMI played an important role in the burden of NCDs, especially CVDs, T2DM and cancers [5,139].

### 4.2. Physical Activity: WHO Recommendations

In addition to instructions for a healthy diet, WHO developed, in 2010, the guidelines “Global recommendations on physical activity for health”, to encourage the increase in PA levels for health benefits, worldwide, and to reduce the burden of overweight, obesity and NCDs [235]. This document, which describes the recommended levels of PA for three age groups (5–17, 18–64 and over 65 years old) has been recently revised: of note, specific recommendations on PA tailored to subpopulations, including pregnant women and persons living with chronic conditions or disability, have been added [240]. Moreover, one year ago, WHO launched the guideline on PA, sedentary behavior and sleep for children under 5 years of age [241]. The basic PA recommendations for health benefits, divided by the three main age groups, are summarized in Table 4 (for more detailed instructions, refer to [235,240]).

The proposed guidelines have been based on solid scientific evidence demonstrating a strong relationship between the type of PA, its duration and intensity and specific health outcomes.

### 4.3. Physical Activity: Effects on Health

Mounting evidence has demonstrated a clear correspondence between frequency, intensity and duration of the PA and the derived benefits, regardless of age, sex, ethnic origin or body weight [242]. Moreover, regular PA has consistently been associated with a reduced risk of mortality [242]. According to the recommended guidelines, either moderate or vigorous intensity activities resulted in nearly the maximum longevity benefit, significantly decreasing the risk of mortality from all causes. No risk has been reported exceeding, even at more than 10 times, the recommended minimum PA [242].

PA impacts the complex physiologic and metabolic networks of our organism, largely affecting cells, tissues, systems and organs. Although many of the specific molecular mechanisms that underlie the response of the body to PA are still under investigations, several beneficial effects on the immune system, mental health, energy and hormonal metabolism, body weight, cardiovascular, respiratory, muscular and osteoarticular systems have been reported. These data clearly indicate the importance of a regular PA in preventing and treating various NCDs [243].

#### 4.3.1. Immune System

The immune system is profoundly influenced by PA: skeletal muscle, in fact, can be considered a major immune regulatory organ, which generates specific proteins, defined myokines, possessing anti-inflammatory and immunoprotective properties [244]. A range of population-level studies have demonstrated that being active for at least 150 min per week is effective against many immune and inflammatory disorders, which reduces the risk of bacterial and latent viral infections [245,246] and supports vaccine responses [247]. Interestingly, these data have been confirmed in a mouse model infected with a lethal dose of influenza virus, showing that moderate exercise improves survival [248].

In particular, it has been demonstrated that regular PA is able to:

1. Stimulate the expression and secretion, by skeletal muscle cells, into the circulation, of anti-inflammatory cytokines (myokines) [249]. Among them, the anti-inflammatory version of IL-6, which leads to the production, by monocytes and macrophages, of regulatory anti-inflammatory mediators (such as IL-10 and IL-1 receptor antagonist) [250].
2. Promote the survival of naive T cells and enhance natural killer (NK) cell production and cytotoxicity, via the expression and release of the muscle-derived cytokine IL-15 [251].
3. Decrease the levels of pro-inflammatory cytokines [252].
4. Produce the meteorin-like protein, which stimulates the conversion of the white adipose tissue into the metabolically active brown adipose tissue and promote M2-macrophage polarization by increasing the secretion of the anti-inflammatory cytokine IL-4 [253,254].

Interestingly, regular PA has been associated with reduced systemic inflammation in the elderly. Evidence demonstrated that reduced PA with age is a major contributor to “immunosenescence”, the age-related immune decline. During the process of aging, a rise in systemic inflammation occurs, namely “inflammaging”, which is associated with an increased risk of developing a range of age-related NCDs [255,256]. Accordingly, it has been demonstrated that the effect of an active lifestyle on older people’s health may impact on inflammaging processes [257].

#### 4.3.2. Cardiovascular Diseases

Several publications have demonstrated that regular PA reduces the risk of CVD mortality, in healthy individuals, by 20–30%, in a dose–response manner [258,259]. The main positive effects of regular PA, particularly aerobic, on the cardiovascular and respiratory systems, are depicted below.

In particular, PA:

1. Increases cardiorespiratory fitness (commonly measured by maximal oxygen uptake, VO_2max_), indirectly decreasing the mortality risks in men and women by 50% and 40%, respectively [260].
2. Improves skeletal muscle oxygen sensing and angiogenesis [243].
3. Ameliorates cardiac output, increasing in turn the body capacity to transport and diffuse oxygen [243].
4. Improves lipid profile, by increasing the high-density lipoprotein (HDL)/low-density lipoprotein (LDL) cholesterol ratio and lowering the plasma triglycerides concentrations [261].
5. Decreases blood pressure [262].
6. Promotes the release of myokines, which engages a crosstalk with adipose-tissue resulting in the reduction in adiposity, increased thermogenesis, lipolytic activity and the conversion of white adipose tissue into the metabolically active brown adipose tissue [263]. Those events contribute to counteract visceral fat accumulation, whose excess is often associated with an increased cardiometabolic risk.

The main characteristics of the cited studies are described in Table 5.

#### 4.3.3. Tumors

Several epidemiological studies have established that PA is associated with a reduced risk of developing cancer. In particular, the WCRF’s update review summarizes evidence from recreational, occupational PA and walking (as a means of transport) on the risk of tumor development. This WCRF report indicates a strong inverse association between the increased PA and the risk of colorectal, endometrial and breast cancer occurrence [5]. The possible mechanisms involved in the inhibition of tumor onset are briefly outlined below.

1. Colorectal cancer: PA induces the reduction in body fatness, insulin resistance and inflammation [264,265,266,267], stimulates digestion and shortens the intestinal transit time [5,268].
2. Endometrial cancer: PA reduces body fatness, circulating estrogen levels, insulin resistance and inflammation, decreases estradiol levels [269], improves insulin sensitivity [270] and reduces chronic inflammation [5,271,272].
3. Breast cancer: PA reduces body fatness, circulating estrogen levels, insulin resistance and inflammation, improves insulin sensitivity, moderates insulin fasting levels as well as IGF-1 secretion [270,272,273], decreases oxidative stress and enhances DNA repair mechanisms [5,274].

Despite some studies demonstrating an inverse correlation between being physically active and the risk of developing esophageal, lung and liver tumors, a deeper validation of these results is still needed [5]. Finally, strong evidence proved that both moderate and vigorous PA indirectly counteract the risk of tumor onset, by decreasing the risk of weight gain, overweight and obesity [5].

#### 4.3.4. Type 2 Diabetes

Widespread evidence from different studies demonstrated that a regular medium to vigorous PA can prevent T2DM. Specifically, regular exercise is able to improve insulin sensitivity, ameliorate glycemic control [275], reduce glycosylated hemoglobin [276], improve body mass composition [277] and increase muscle glucose uptake through an insulin-independent mechanism [278,279]. In agreement with that, tailored PA recommendations for T2DM or pre-diabetes patients improve blood glucose control, reduce cardiovascular risk factors, decrease body fat percentage and increase lean body mass [279].

#### 4.3.5. Neurodegenerative Diseases

Several studies have supported PA as a therapy for mental health improvements in cognition [280,281], neurodegenerative pathologies (Alzheimer’s and Parkinson’s disease) [282], anxiety [283] and depression [284,285]. The improvement in psychological and neurological parameters may depend on the local and systemic increased expression of neurotrophins, such as the brain-derived neurotrophic factor (BDNF) [286,287], together with the modulated expression of neurotransmitters and hormones [288], the improved cerebral blood flow and reduced neuroinflammation [289,290].

#### 4.3.6. Microbiota

Some human interventional studies have examined the effects of PA on gut microbiota, demonstrating that exercise both qualitatively and quantitatively changes gut microbial composition and function, with several benefits for the host, including enriching microbiota diversity towards more “health-associated” microbes. These bacteria are able to modulate mucosal immunity, improving barrier functions and stimulate the production of substances (SCFAs) that protect against gastrointestinal disorders and improve performance [291,292,293,294,295,296,297].

However, future research is needed to provide deeper understanding on the molecular mechanisms, triggered by PA, that determine changes in the composition and functions of the gut microbiota and their health-related effects.

#### 4.3.7. Aging

Recent studies reported evidence suggesting that the health benefits of PA may be mediated through an effect on aging mechanisms. Several correlations between PA and biomarkers of biological age have been found: in particular, PA showed a beneficial effect on the rate of epigenetic aging [298,299]. Furthermore, several observational studies have shown a relationship between PA levels and telomere length: moderate levels of PA have been associated with longer telomeres in senility compared to low or very high levels of activity, suggesting a dose-dependent effect on telomere length [300,301]. Senescent cells, despite being proliferatively quiescent, are highly metabolically active, contributing to the aging process in many ways, including the secretion of pro-inflammatory cytokines. In mice, senescent cell removal has been shown to prevent age-related diseases and increase lifespan [302]. In addition, senescent cells accumulate in adipose tissue: interestingly, it has been demonstrated that exercise can both prevent the accumulation of these cells in a mouse model of diet-induced obesity [303] and, in humans, it can lower fat mass, reducing the infiltration of inflammatory monocytes to adipose tissue and increase the polarization of resident macrophages to an M2-like, anti-inflammatory, phenotype [253].

Notably, when impact exercise is combined with moderate or high-intensity progressive resistance activity, an improved bone mass might be observed, resulting in the prevention of osteoporosis [304]. Importantly, PA performed in youth increases peak bone mass and could reduce the burden of fractures [305].

In conclusion, PA may be able to counteract some of the mechanisms associated with senescence, ameliorating most of the typical aging phenotypes.

#### 4.3.8. Overweight and Obesity

As extensively discussed above, moderate to vigorous PA is an important lifestyle behavior that contributes to the maintenance of a healthier body composition, weight management and prevention of weight gain. However, PA alone shows little or no long-term effect on body weight loss. Mounting evidence has demonstrated that, in order to maintain long-term weight loss and prevent a weight regain, it is crucial to couple PA with healthy dietary intervention [306,307,308]. On the other hand, a healthy diet alone will be less effective for both short- and long-term weight control, thus suggesting that the best approach to optimize the regulation and composition of body weight must involve both those key lifestyle elements.

Furthermore, as previously discussed and outlined below in Table 6, PA elicits numerous health benefits and hence should be always encouraged, regardless of body size (Table 6).

## 5. Combined Effects of Mediterranean-Type Dietary Pattern and Physical Activity

Mounting evidence demonstrated that the association between a Mediterranean-type dietary pattern and PA results in several health benefits, especially on the cardiovascular system.

### 5.1. Cardiovascular Health

Studies on the combined effects of MeD and PA on cardiovascular health started in the early 2000s: it has been shown that the adoption of a MeD by physically active individuals significantly reduced the coronary risk and significantly prevented the occurrence of acute CHD in controlled hypertensive subjects [309]. Moreover, obese women following a hypoenergetic MeD and an exercise program showed improved cardiovascular disease risk factors, together with a preserved body cell mass, after 4 months of treatment [310]. The “ATTICA” epidemiological study (performed in Attica—Athens metropolitan region, Greece), applied to a sample of more than 3000 free-living individuals, revealed that increased PA, in combination with a strict adherence to the MeD, was associated with improved total antioxidant capacity levels, essential to protect the cardiovascular system [311]. The effects of combined MeD and exercise intervention on lower- and upper-limb cutaneous microvascular functions in an older healthy population (aged 55 ± 4 years), showed, after 8 weeks, an improvement in the age-related microcirculatory endothelial dysfunction and an increase in exercise tolerance, thus reducing the cardiovascular risk [312]: of note, these improvements were still evident one year after this intervention, suggesting that, in this high-risk group, also a brief treatment combining MeD with PA might result in long-term cardiovascular health benefits [313]. Moreover, a small group of postmenopausal women (aged 54.6 ± 3.6 years) were randomized into either exercise training only or exercise training combined with MeD, for eight-weeks: combination of MeD with regular PA resulted in additional improvement in the microcirculatory vascular function, thus expecting a further cardiovascular risk-reduction [314]. In addition, a 3-year prospective controlled trial randomized on adult subjects with MetS, showed that MeD and regular aerobic exercise program significantly improves abdominal circumference, blood pressure and HDL cholesterol levels [315]. Moreover, the association of MeD and exercise program, in subjects with known CHD, not only decreased cardiovascular risk, but also showed additional benefits in reducing protein, cholesterol intake and abdominal fat [316]. Recently, more than 19,000 participants from a prospective cohort were followed up for more than 10 years: subjects having a higher level of adherence to the traditional MeD and adopting an active lifestyle showed a 75% relatively reduced risk of cardiovascular disease [317]. In another cohort of patients affected by atrial fibrillation, the combination of good adherence to MeD with a high level of regular PA resulted in a lower risk of silent brain infarct. Of further interest, when diet and PA were analyzed as independent variables, no statistically significant association was found [318]. In line with these data, a very recent study analyzing the effectiveness of a personalized MeD and a PA intervention in a population of women at risk of CVDs demonstrated that the combination of the two treatments led to body weight loss, body composition remodeling and cardiovascular risk index reduction [319].

### 5.2. Metabolic Alterations

MeD and PA interventions can also be applied to counteract abnormal metabolic processes. Indeed, the combination of MeD and an active lifestyle resulted in the reduction in the risk and severity of non-alcoholic fatty liver disease [320]. The association of MeD and mild PA modifications also showed positive outcomes in a pilot study performed on individuals affected by psychiatric disorders: after 6 and 12 months of treatment, patients’ metabolic and anthropometric parameters were improved, reducing the risk for MetS, cardiovascular diseases and other complications [321]. Moreover, a systematic review and meta-analysis of randomized controlled trials showed that the adhesion to a MeD and PA combined program could provide a reduction in the metabolic risk in adults [322]. More recently, a 12 month, randomized, single-blinded, diet-controlled study, performed on 124 obese patients, evaluated and compared the effects of an energy-restricted MeD or a standard hypolipemic diet, both associated with PA, on metabolic syndrome parameters: interestingly, although both diets yielded similar weight reduction results, when combined with PA, only adherence to the MeD plus PA was associated with more prominent decrease in MetS parameters [323].

A randomized 12-week intervention on 40 MetS patients (aged 50–66 years) showed that a hypocaloric MeD associated with moderate-to-high-intensity training was more efficient, than MeD alone in triggering body weight loss and ameliorating insulin sensitivity, triacylglycerols and blood pressure. Moreover, cardiorespiratory fitness and ischemic reactive hyperemia were improved and the number of endothelial progenitor cells increased. These results indicate that the combined effect of MeD and PA is able to ameliorate both the regenerative capacity of endothelium and the fitness of MetS patients [42]. A recent systematic review of observational studies demonstrated that a Mediterranean-like dietary pattern, together with PA, before or in early pregnancy, were associated with lower risks of developing gestational diabetes mellitus [324]. Finally, a PREDIMED-Plus trial, performed on more than 600 overweight/obese patients with MetS (aged 55–75 years), reported that 12 months of weight loss lifestyle intervention, based on an energy-restricted MeD and PA promotion, was able to effectively decrease adiposity and cardiovascular risk factors in overweight/obese older patients with MetS. Moreover, in the same study, it was demonstrated that this intervention caused modest, but potentially important, improvements in glycemic control, insulin sensitivity and dyslipidemia in participants with or at risk for T2DM [325].

### 5.3. Bone and Muscle Health

The combination of MeD and PA has also been demonstrated to be effective for the prevention of bone and muscle disease. In a randomized clinical trial performed on 106 women with rheumatoid arthritis, patients were assigned to MeD plus PA, PA alone and MeD alone groups. After 24 weeks, a disability improvement was observed only in the group treated with MeD plus PA, together with increased hand grip strength and decreased weight and waist circumferences [326]. In 2020, another randomized clinical trial, including 144 women with rheumatoid arthritis, demonstrated that a dynamic PA program, combined with MeD, improved the quality of life in patients with low disease activity treated with conventional disease-modifying antirheumatic drugs [327]. A cross-sectional analysis with 956 adolescents (aged 12–18 years) demonstrated that high PA and optimal adherence to MeD positively associated with a higher quality of muscular fitness [328]. Consistently, another study performed on 13 healthy subjects, following a 3-month protocol combining MeD and PA, showed an improved posture in terms of realignment and the rebalancing of body segments [329]. Finally, a 6-month randomized controlled parallel-group, single-blinded clinical trial, performed on Alzheimer’s diseases patients, is in progress, in order to monitor after 1, 3 and 6 months of follow-up, how the combination of a physical exercise program with a MeD will affect bone mineral density, gait, balance and fall risk [330].

### 5.4. Neurological Health

A combined effect of MeD and PA has also been observed on neurological health. A six-month study performed on obese women (aged 46.31 ± 4.07 years), following a hypocaloric MeD and a PA program, showed a modified functional connectivity between the brain structures involved in the pathophysiology of obesity, demonstrating how the combination of these two interventions can be effectively applied in weight loss programs [331]. In addition, a randomized controlled trial is in progress in Australia, investigating the effects of MeD and PA on cognitive performance in healthy older people (age 60–90 years) living independently. Moreover, this study also aims to investigate the possible biological mechanisms involved in this process [332]. Considering the increasingly aging society, being able to ameliorate the rate of cognitive decline in older people through feasible lifestyle changes may be of substantial importance to public health.

### 5.5. Aging

Telomere length is a predictive biomarker of premature aging. Accordingly, telomere shortening has been linked to age-related diseases and NCDs. As already discussed previously, both MeD and PA are independently able to preserve telomere lengths. Not surprisingly, the combination of a healthy Mediterranean-like diet with regular PA reduces inflammation and oxidative stress levels, lowering telomere-shortening rate thus, indirectly, decreasing the risk of NCD and aging-associated disease occurrence [333].

### 5.6. All-Cause Mortality

In agreement with all the evidence previously described, combining the effect of a MeD-like pattern with a regular PA reduces overall mortality. A large prospective study, which analyzed data from more than 19,000 participants, demonstrated that MeD and PA had multiple effects in the reduction in overall mortality risk [334]. Another prospective study, performed on more than 7000 older adults with high vascular risk (aged 67 ± 6.2 years), showed that higher levels of leisure-time PA, regardless of the intensity, and strict adherence to MeD, were both separately and jointly associated with reduced all-cause mortality [335]. Consistently, the Melbourne Collaborative Cohort Study, which followed more than 22,000 participants for approximately 14 years, showed that the combination of adherence to a Mediterranean-style diet and high PA resulted in an estimated reduction in all-cause mortality [336].

## 6. Conclusive Remarks

In recent years, a significant increase in NCD burden has been globally observed and this escalation is expected to rise even faster in the future, especially in the developing world. Accounting for the greatest share of early death and disability worldwide, those pathologies have devastating effects on the global economy. Thus, investing in NCDs prevention and management represents an ethical and economic obligation, which could avert many premature deaths and prevent severe economic losses. A healthy diet and a regular physical program are among the most cost-effective, affordable and efficient behavioral interventions able to prevent and manage the burden of NCDs. Mounting evidence, from many studies performed across different countries, indicates that a Mediterranean-type dietary pattern can efficiently prevent and control the onset of the major NCDs, reduce the overall mortality and promote a healthy aging. Similarly, PA has consistently been associated with a reduced risk of mortality, due to its capability to counteract and treat NCDs. Although preliminary, studies analyzing the combination of a Mediterranean-type dietary pattern and PA have shown significant amelioration of several biomarkers associated with NCD development. Based on this promising scientific evidence, the adoption of a Mediterranean-like dietary pattern, combined with PA, should be more and more encouraged. However, further studies are needed, in order to identify the biological and physiological mechanisms able to explain how specific nutritional and PA interventions can enable people to live a longer and healthier life. In addition, the “Omics” technologies, which rapidly generate an enormous amount of data, may contribute to extensively investigate multiple pathways specifically activated and stimulated by PA and nutritional interventions. These progresses are long expected, in order to promptly counteract the onset of major NCDs and develop individualized health programs, which include tailored dietary plans and specific PA interventions.

## Figures and Tables

**Table 1:** Main characteristics of some of the indexes used to estimate the adherence to the Mediterranean diet (MeD). For a deeper review, see [50,51,56].

Index	Components	Score	Range of Adherence
Mediterranean Diet Score 1 (1995) (MDS-1) [53]	8 (7 food groups/foods, 1 ratio)	0–1	0–8
Mediterranean Diet Score 2 (2003) (MDS-2) [55]	9 (7 food groups/foods, 1 nutrient, 1 ratio)	0–1	0–9
Mediterranean Diet Score (2001) (MD Score 01) [57]	8 (6 food groups/foods, 1 nutrient, 1 ratio)	0–1	0–8
Mediterranean Diet Score (2004) (MD Score 04) [58]	9 (9 food groups)	1–3	9–27
Mediterranean Dietary Pattern (2002) (MDP 02) [59]	8 (6 food groups/foods, 2 nutrients)	0–5	5–40
Dietary Score (DS) [60]	11 (11 food groups/foods)	0–5	0–55
Mediterranean Adequacy Index (MAI) [61]	16 (16 food groups/foods)	-	0–100
Mediterranean food pattern PREDIMED (Prevención con Dieta Mediterránea—Prevention with Mediterranean Diet) Study (MeDiet-PREDIMED/MEDAS—Mediterranean Diet Adherence Screener) [62]	14 (14 food groups/foods)	0–1	0–14
Mediterranean-Style Dietary Pattern Score (MSDPS) [63]	13 (13 food groups/foods)	0–10	0–100
Mediterranean Lifestyle (MEDLIFE index) [64]	28 (21 food groups/foods, 1 nutrient, 6 lifestyle factors)	0–1	0–28
Relative Mediterranean Diet Score (rMED) [65]	9 (8 food groups/foods, 1 nutrient)	0–2	0–18
Italian Mediterranean Index (ITALIAN-MED) [66]	11 (11 food groups/foods)	0–1	0–11

**Table 2:** Characteristics of studies describing the effects of a Mediterranean diet on cardiovascular diseases (CVDs).

Type of Study	Number of Participants	Primary Outcomes	Year of Publication
Clinical trial	11,323	Reduction in mortality after myocardial infarction	2003 [67]
Multi-center, prospective cohort study	519,978	Reduction in mortality among coronary patients Reduction in overall mortality among apparently healthy individuals	2007 [68]
Cohort study	40,011	Adherence to a Mediterranean style diet inversely associates with total CVD, in particular with fatal CVD	2012 [69]
Literature review	-	Analysis of the effects of whole and parts of MeD, with regard to population-based and experimental data, highlighting CVD morbidity, mortality and CVD surrogates	2015 [70]
Prospective cohort design	25,994	Risk reduction in CVD events	2018 [71]
Parallel-group, multicenter, randomized trial	7447	Reduction in incidence of major CV events in persons at high CV risk	2018 [72]
Systematic review and meta-analysis of observational studies	-	Protective effect of MeD against the risk of CVD	2019 [73]
Literature review	-	Role exerted by foods, commonly consumed in the Mediterranean area, in prevention and progression of different types of CVDs and cancer	2019 [23]
Article (brief communication)	-	Oleocanthal, contained in extra virgin olive oil, has an Ibuprofen-like activity	2005 [74]
Multicenter, randomized, controlled, clinical trial	7216	Extra virgin olive oil consumption is associated with a reduced risk of CVD and mortality in a Mediterranean population at high CV risk	2014 [75]
Systematic review and meta-analysis of randomized controlled trials	-	MeD decreases inflammation and improves endothelial function	2014 [76]
Randomized controlled trial	612	Polyphenol consumption could exert a protective effect against some CV risk factors	2016 [77]
Umbrella review of meta-analyses of randomized controlled trials	-	Beneficial effect on anthropometric parameters and cardiometabolic risk factors	2020 [78]

**Table 3:** Major Mediterranean-type dietary components and their effects on health.

Dietary Components	Effects on Health
Unsaturated fats: MUFAs and PUFAs (extra virgin olive oil, nuts, seeds, omega-3 rich fish)	(i) (Antioxidant, anti-inflammatory and antiatherogenic properties, resulting in cardioprotective actions [72,74,75,76,77]. (ii) Protective effects against cancer development and progression: preventing DNA damages, counteracting cell degeneration, proliferation and metastasis [99,119,120]. (iii) Decreased biomarkers of subclinical inflammation and increased levels of adiponectin, an anti-inflammatory cytokine inversely associated with T2DM risk [133,134]. (iv) Beneficial effects on insulin sensitivity [137]. (v) Protection against the age-related cognitive decline [47,48], some benefits towards the onset of Alzheimer’s [49] and, to a lesser extent, Parkinson’s diseases [44]. (vi) Maintenance of low levels of serum inflammatory markers beneficial for the microbiota [166,167]. (vii) Delay of aging processes and the promotion of healthy longevity [178,179].
Fibers and phytosterols (whole grains, legumes, nuts, vegetables, fruits, extra virgin olive oil)	(i) Antioxidant activities and antiatherogenic properties, resulting in cardioprotective actions [72,74,75,76,77]. (ii) Protective effects against cancer development and progression: preventing DNA damages, counteracting cell degeneration, proliferation and metastasis [99,119,120]. (iii) Decreased biomarkers of subclinical inflammation and increased levels of adiponectin, an anti-inflammatory cytokine inversely associated with T2DM risk [133,134]. (iv) Maintenance of low levels of serum inflammatory markers beneficial for the microbiota [166,167]. (v) Delay of aging processes and promotion of healthy longevity [178,179].
Fibers (whole grains, legumes, vegetables, fruits, nuts)	(i) Satiety induction, caloric intake reduction, weight gain prevention [135]; delay in glucose absorption, which decreases the concentration of fasting blood glucose and insulin [136]. (ii) A 20% reduced risk of diabetes [131]. (iii) Elevated concentration of SCFAs with anti-inflammatory and antitumor effect, in particular toward CRC [152].
Phytosterols (extra virgin olive oil)	(i) Beneficial effect on bone health [150].

**Table 4:** WHO recommended levels of physical activity (PA) for three age groups: 5–17 years old, 18–64 years old and 65 years old and above [235].

Age Group	Recommended Levels of Physical Activity
Children and youth aged 5–17	(i) Children and youth aged 5–17 should accumulate at least 60 min of moderate to vigorous intensity daily PA [235]. (ii) Doing more than 60 minutes per day of PA provides additional health benefits [235]. (iii) Most of the daily PA should be aerobic. Vigorous intensity activities should be considered, including those that strengthen muscles and bones, at least 3 times per week [235].
Adults aged 18–64	(i) Adults aged 18–64 should either do, at least, 150 min of moderate intensity aerobic PA throughout the week, or do, at least, 75 min of vigorous intensity aerobic PA throughout the week, or an equivalent combination of moderate and vigorous intensity activity [235]. (ii) Aerobic activity should be performed in bouts of at least 10 min duration [235]. (iii) For additional health benefits, adults should increase their moderate intensity aerobic PA to 300 min per week, or engage in 150 min of vigorous intensity aerobic PA per week, or an equivalent combination of moderate and vigorous intensity activity [235]. (iv) Muscle-strengthening activities should be performed involving major muscle groups on 2 or more days a week [235].
Adults aged 65 years and above	(i) Adults aged 65 years and above should do at least 150 min of moderate intensity aerobic PA throughout the week, or do at least 75 min of vigorous intensity aerobic PA throughout the week, or an equivalent combination of moderate and vigorous intensity activity [235]. (ii) Aerobic activity should be performed in bouts of at least 10 min duration [235]. (iii) For additional health benefits, adults aged 65 years and older should either increase their moderate intensity aerobic PA to 300 min per week, or engage in 150 min of vigorous intensity aerobic PA per week, or an equivalent combination of moderate and vigorous intensity activity [235]. (iv) Adults of this age group, with poor mobility, should perform PA to enhance balance and prevent falls on 3 or more days per week [235]. (v) Muscle-strengthening activities should be performed involving major muscle groups, on 2 or more days a week. When adults of this age group cannot do the recommended amounts of PA, due to health conditions, they should be as physically active as their abilities and conditions allow [235].

**Table 5:** Characteristics of studies describing the effects of physical activity on CVDs.

Type of Study	Number of Participants	Primary Outcomes	Year of Publication
Large pooled cohort analysis	>650,000	More leisure time PA was associated with longer life expectancy across a range of activity levels and body mass index (BMI) groups.	2012 [258]
Prospective, observational cohort study	55,137	Running, even 5–10 min/day and at slow speeds (<6 miles/h), is associated with markedly reduced risks of death from all causes and CVD.	2014 [259]
Literature review	-	Analysis of the association between volume of PA, health, CV and overall mortality.	2012 [260]
Literature review	-	Effects of endurance exercise training on CV fitness and general health outcomes. Description of the molecular connections from endurance training to mental health. Analysis of the relationships between T2DM, mitochondria and endurance training.	2018 [243]
Meta-analysis of randomized controlled trials	-	Overall results suggest that aerobic exercise lowers LDL in adults with T2DM.	2017 [261]
Randomized controlled trial	36	Prolonged exercise is more effective than multiple short sessions to reduce the risk of MetS and the atherogenic index in middle-aged obese women.	2017 [262]
Literature review	-	Analysis of the latest knowledge on myokines and muscle-adipose tissue crosstalk.	2018 [263]

**Table 6:** Major physiological effects of physical activity and its outcomes on non-communicable diseases (NCDs) prevention and managing.

Immune system	(i) Stimulation of the expression and secretion of myokines [249], among them, the anti-inflammatory version of IL-6 [250]. (ii) Promotion of the survival of naive T cells and enhancement of natural killer (NK) cells production and cytotoxicity [251]. (iii) Decrease in the levels of pro-inflammatory cytokines [252]. (iv) Production of the meteorin-like protein, which stimulates: white to brown adipose tissue conversion, increased secretion of IL-4 and macrophage polarization from an M1-like to an M2-like anti-inflammatory phenotype [253,254].
Aging	(i) Beneficial effect on the rate of epigenetic aging [298,299]. (ii) Preservation of telomere length [300,301]. (iii) Promotion of the removal of senescent cells from adipose tissue [302,303]. (iv) Reduction in fat mass, decrease in inflammatory monocyte infiltration into adipose tissue and increase in resident macrophage polarization, from an M1-like to an M2-like phenotype [253]. (v) Optimization of peak bone mass in youth, maintenance and improvement of bone mass in adults and elderly [304,305].
Microbiota	(i) Modification, both qualitatively and quantitatively, of gut microbial composition and function [291,294,295,296,297]. (ii) Stimulation of bacteria growth, able to modulate mucosal immunity [291,294,295,296,297]. (iii) Preservation of intestinal barrier functions [291,294,295,296,297]. (iv) Production of substances able to protect against gastrointestinal disorders [291,294,295,296,297].
Cardiovascular Diseases	(i) Increase in cardiorespiratory fitness [260]. (ii) Improvement of skeletal muscle oxygen sensing and angiogenesis [243]. (iii) Amelioration of cardiac output [243]. (iv) Increase in the HDL/LDL ratio and decrease in triglycerides plasma concentrations [261]. (v) Decrease in blood pressure [262]. (vi) Reduction in adiposity, increase in thermogenesis, conversion of white adipose tissue into the metabolically active brown adipose tissue and increase in lipolytic activity [263].
Tumors	Colorectal cancer: (i) Reduction in body fatness, insulin resistance and inflammation [264,265,266]. (ii) Promotion of digestion and improvement of intestinal transit time [5,267]. Endometrial cancer: (i) Reduction in body fatness, circulating estrogen levels, insulin resistance and inflammation [269]. (ii) Decrease in estradiol levels [268]. (iii) Improvement of insulin sensitivity [269]. (iv) Reduction in chronic inflammation [5,270,271]. Breast cancer: (i) Reduction in body fatness, circulating estrogen levels, insulin resistance and inflammation [270,272,273]. (ii) Improvement of insulin sensitivity, reduction in insulin fasting levels and IGF-1 secretion [270,272,273]. (ii) Reduction in oxidative stress and enhancement of DNA repair mechanisms [5,274].
Type 2 diabetes	(i) Improvement of insulin sensitivity and glycemic control [275]. (ii) Reduction in glycosylated hemoglobin [276]. (iii) Improvement of body mass composition [277]. (iv) Increase in muscle glucose uptake [278,279]. (v) Improvement of blood glucose control, reduction in cardiovascular risk factors, decrease in body fat percentage and increase in lean body mass [279].
Neurodegenerative Diseases	(i) Improvement of cognition [280,281], neurodegenerative pathologies (Alzheimer’s and Parkinson’s disease) [282], anxiety [283] and depression [284,285]. (ii) Increase in local and systemic expression of neurotrophins [286,287]. (iii) Modulation of neurotransmitter and hormone expression [288]. (iv) Improvement of cerebral blood flow and reduction in neuroinflammation [289,290].
Overweight and obesity	(i) Reduction in adiposity, increase in thermogenesis, conversion of white adipose tissue into the metabolically active brown adipose tissue and increase in lipolytic activity [253,263]. (ii) Improvement of body mass composition [277]. (iii) Contribution to elicit and maintain a long-term weight loss and prevent weight regain (in combination with diet) [306,307,308].

## Data Availability

Data sharing not applicable. No new data were created or analyzed in this study. Data sharing is not applicable to this article.
